# Morphological and molecular basis of ovarian serous carcinoma

**DOI:** 10.1016/S1674-8301(10)60036-X

**Published:** 2010-07

**Authors:** Daniel G Rosen, Zhihong Zhang, Weiwei Shan, Jinsong Liu

**Affiliations:** a Department of Pathology, the University of Texas M.D. Anderson Cancer Center, Houston, TX 77030, USA; b Department of Pathology, the First Affiliated Hospital of Nanjing Medical University, Nanjing 210029, Jiangsu Province, China

**Keywords:** ovarian carcinoma, grading, morphology, molecular genetics, tumorigenesis

## Abstract

Serous carcinoma is the most common type of epithelial ovarian cancer. In this review, we provide a comprehensive picture of ovarian serous cancers from multiple aspects: the first part of this review summarizes the morphological, histological, and immunological signatures of ovarian serous carcinoma; subsequently, we review the history of the evolvement of different grading systems used in ovarian serous cancer; in the end, we focus on characterizing the genetics that underlie the 2-tiered pathways through which ovarian serous cancers are believed to arise: the low-grade and the high-grade pathways.

## INTRODUCTION

Ovarian epithelial carcinoma is the second most frequent type of cancer in the female genital tract, and the most lethal gynecological malignancy[1]–[3]. It is estimated that in 2010 a total of 21,880 new ovarian cancer cases will be diagnosed and 13,850 will die of this disease in the USA[4]. Ovarian carcinoma encompasses a heterogeneous groups of disease with several different isotopes. Among them, serous carcinoma is the most common type and accounts for more than 45%[5],[6] of malignant epithelial ovarian tumors. In this review we summarize recent advances in the pathology of ovarian serous cancers, and discuss the molecular and genetic evidence supporting the 2-tiered developmental systems of ovarian serous cancers.

## MORPHOLOGIC FEATURES

### Gross appearance

Serous carcinoma of the ovary varies greatly in size ranging from microscopic to over several kilograms. The external surface of the lesion may be smooth, coarse or sometimes entirely exophytic showing papillary structures denoting a serous surface carcinoma. High grade serous carcinomas often show solid, multiloculated cystic areas signified by necrosis, friability and hemorrhage. On clinical presentation, ovarian serous carcinoma is usually a large mass with bilateral presentation in the ovary in two-thirds of cases. Omental metastases are often present consisting of white or gray confluent nodules known as “omental cake”.

### Histology

#### Low-grade serous carcinoma

**Fig. 1:**
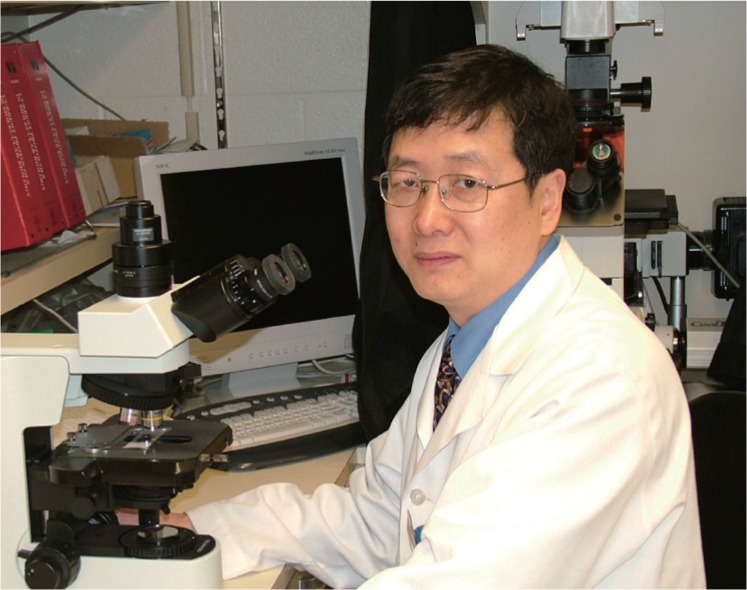
The morphology of ovarian serous carcinoma. A: low power view (×100) of low grade serous carcinoma (up left) arising from micropapillary low grade serous carcinoma (low right). B: high power view (×400) showing uniform nuclei of low grade serous carcinoma. C: low power view (×100) of low-grade serous carcinoma. D: high power view (×400) of high grade serous carcinoma showing marked nuclear atypia.

Low grade serous carcinomas are usually characterized by a papillary growth occupying a variable extent of a cystic lining. The lining cells show minimal nuclear atypia (***Fig. 1A and 1B***). It is very common to see cellular buds without fibrovascular stroma that appear unattached to the main papillae. The presence of frank destructive invasion (>3.0 mm) of the ovarian stroma is required to differentiate low-grade serous carcinomas from serous tumors of low malignant potential. Signs of invasion can be determined by the presence of a desmoplastic reaction of the stroma with variable degrees of lymphocytic inflammatory infiltration into surrounding small nests of tumor cells, and necrosis and hemorrhage are usually absent. In rare occasions, only a solid growth pattern is identified without a cystic component. In this case the cytologic atypia of the neoplastic cells is helpful to distinguish low-grade serous lesions from high grade lesions. The lining cells in low grade serous carcinoma show uniform nuclei with mild size variation, uniform chromatin pattern and small conspicuous nucleoli, while high grade serous carcinoma show marked nuclear atypia (see below). Mitotic figures are scant, usually less than 12 mitosis/10 high-power fields (HPFs) (mean is 4 mitosis/10 HPFs)[7]. Psammoma bodies, which are small, whorled calcifications, may be present.

#### High grade serous carcinoma

High-grade serous carcinoma is often composed by both a complex papillary pattern and a solid pattern of serous cells with marked nuclear atypia (***Fig. 1C and 1D***). In general, the pattern is a mixture of cystic, papillary, and solid growth, but it is not unusual to find one pattern more predominant than others. Extensive cellular budding, obvious nuclear atypia and diffuse stromal invasion are common in solid growth areas. Laminated psammoma bodies can be present but in a less extent than those in low-grade serous carcinoma. Isolated bizarre tumor giant cells are commonly seen. In some occasions, multi-nucleation can be present, and cells are positive for human chorionic gonadotropin (hCG) resembling syncytiotrophoblast cells. Mitosis, including abnormal mitosis, are usually numerous (>12 mitosis/10 HPFs), and necrosis is often extensive. Less common of a gland-like pattern can be seen mimicking endometrioid type carcinoma of the ovary. Typically, these glands are composed of irregular slit-like spaces. More rarely, high-grade serous carcinomas can mimic adenoid cytic carcinoma, undergoing squamous differentiation, having microcysts, or having a focal reticular pattern of a yolk sac tumor.

## IMMUNO-PROFILE

Immunohistochemically, serous carcinomas are positive for cytokeratin 7 (100%)[8], CAM 5.2 (100%), epithelial membrane antigen (100%)[9], B72.3 (92%), WT-1 (100%), p53 (81%), and CA-125 (91%)[10],[11]. Other markers less commonly expressed are cytokeratin 20 (34%), vimentin (45%), S-100 (30%) and carcinoembryonic antigen (19%). Serous carcinomas are consistently negative for calretinin and other mesothelial markers[10]. About half of the cases of ovarian serous cancers are positive for estrogen, progesterone, and/or androgen receptors[12].

## GRADING SYSTEMS

Many studies have reported that the grade of ovarian carcinoma is an important prognostic factor and therapeutic implication[13]–[17]. However, there is no agreement among pathologists regarding a uniform histological grading system for ovarian serous carcinoma. Current grading systems lack consistent criteria and have poor reproducibility. For example, the FIGO system[18] is based on architectural features; the WHO system[19] is based on the observer's impression of architectural and cytologic features; the Silverberg's system[20] is based on the architectural pattern, cytologic atypia, and mitotic index; the M.D. Anderson Two-Tier System[7] is based on nuclear atypia and the mitotic rate; the Bichel/Jakobsen system[21] is based on architectural features, cytologic features, mitotic index, invasive mode, capsular penetration, and vascular invasion; the Broder's system[22] is based on the architectural differentiation and degree of cytologic atypia. Here we review the four most commonly used systems for grading ovarian serous carcinomas.

### The FIGO grading system

In 1971, the FIGO system emphasized on the classification and staging of the female pelvis tumors, showing an important impact on prognosis. The proposed grading system of ovary was similar to that of the uterine endometrial cancer[23], and was based on architectural features[18]. This system has been widely used for a long time and is still in use by some institutions. The grading is defined by the ratio of glandular or papillary structures *vs* solid tumor growth pattern. In this system, Grade 1 is defined as having less than 5% of solid tumor growth; Grade 2 is having 5% to 50% of solid tumor growth, and Grade 3 is equivalent to having more than 50% solid tumor growth[20]. Multiple investigators[14],[15],[24],[25] used the FIGO grading system in their studies. However, it can be quite subjective in term of defining these so called solid areas.

### The World Health Organization grading system

The WHO grading system[19] recommended a three-tiered grading system. It takes into account histo-architectural features and cytologic atypia. As such, the WHO grading system is a very subjective one relying on the observer's experience without quantitative measurement[15],[20],[26]. Like FIGO system, WHO system distinguishes between low, intermediate, and high grade serous cancers using an “intuitive” method[27]. The architectural pattern is evaluated as it is in the FIGO system, and cytologic features are judged by the observer's impression of the degree of cellular differentiation, such as mild, moderate or poor differentiation. Several authors[13],[28] reported using this system in their articles.

### The Silverberg's group system

Silverberg's group[15],[20] suggested a grading system in analogy to one used in grading mammary carcinomas. They found that their system had valuable prognostic information and could be used in all histological types of cancers. This system relies on assessments of architectural pattern, nuclear atypia, and mitotic activity. The tumors are graded as following: architectural pattern (predominant): glandular pattern scores 1 point, papillary pattern scores 2 points, and solid pattern scores 3 points; nuclear pleomorphism: slight atypia scores 1 point, moderate atypia scores 2 points, and marked atypia scores 3 points; mitotic activity (in most active region count mitotic figures/10HPFs): up to 9/10HPFs scores 1 point, 10-24/10HPFs scores 2 points, and >25/10HPFs scores 3 points. When the 3 numbers from these 3 aspects are combined, Grade 1 is defined as a total score equal to 3-5 points, Grade 2 to 6-7 points, and Grade 3 to 8-9 points. The total score separates cancers into well differentiated (G1), moderately differentiated (G2), and poorly differentiated (G3) groups. As mentioned above, this system is simplified and highly reproducible compared with the others. In a study carried out by Mayr *et al*[29], the Silverberg's grading system was tested on a series of 192 ovarian carcinomas and provided evidence for the validity of this grading system. Sato *et al*[30] applied this system to 70 cases of ovarian carcinomas and obtained similar results. Both authors showed that this grading system can be easily applied to different types of ovarian cancers excluding ovarian clear cell carcinoma including mestastatic carcinoma[31].

### Two-Tier System

The two-tier grading system[7] for ovarian serous carcinoma was developed by researchers in the M.D. Anderson Cancer Center and has been in use at the M.D. Anderson Cancer Center for more than 10 y with excellent results. The two-tier grading system is based primarily on the assessment of nuclear atypia and the mitotic rate of cancer cells. Unlike other grading systems mentioned above, this system consists of two groups: grade 1 (low-grade) and grade 2 (high-grade), and only evaluates ovarian serous carcinoma. Nuclear features of “low-grade” serous cancers are defined as mild to moderate atypia with uniformly round or oval nuclei. On the other hand, nuclear features of the “high-grade” counterparts are characterized as pleomorphism by variation 3:1 in nuclear size and shape with macronucleoli. The mitotic index (in the most mitotically active area of the tumor) is evaluated as such: if there are less than 12 mitotic figures/10 HPFs, the cancer belongs to the low-grade group; if more than 12 mitotic figures/10 HPFs, the specimen is a high-grade cancer. This system has good overall correlation to the Silverberg and FIGO systems, is user-friendly, and appears to have good reproducibility[7].

These four common grading systems are summarized in ***Table 1***. It is interesting that many published articles have come to the same conclusion with regard to the prognostic significance of histopathology grade despite the use of different grading systems. However, recent advance in molecular genetics demonstrated that ovarian serous carcinoma developed along two distinct pathways: the low-grade and the high-grade pathways. Thus, as is detailed below, genetic analysis of ovarian serous cancers further supports the practicality of the two-tier system but not other grading systems developed at M.D. Anderson Cancer [32].

**Table 1:** The four most commonly used grading systems for ovarian serous cancer

System	Define	Gl	G2	G3
FIGO	Solid growth pattern	<5%	5%∼50%	>50%
WHO	Architectural pattern and Cytologic atypia	High differentiation	Intermediate differentiation	Low differentiation
Silverberg's system	Architectural pattern: (score)	Total score	Total score	Total score
	Glandular(l),papillary(2),solid(3)	3-5	6-7	8-9
	Cytological atypia: (score)			
	Slight(l),moderate(2),marked(3)			
	Mitotic figures (score)			
	0-9/10HPFs(l), 10-24/10HPFs(2), >25/10HPFs(3)			
Two-tier system		Low-grade	High-grade	
	Nuclear atypia	Mild to moderate	Severe	
	Mitotic figures	< 12/10 HPFs	> 12/10 HPFs	

## GENETIC FEATURES

### Genetic features of low-grade serous carcinoma

***Kirsten RAS oncogene homolog* (*KRAS*):** *KRAS* is a member of the mammalian *RAS* gene superfamily that encodes a small protein GTPase. *KRAS* is one of the best-documented oncogenes, and is frequently activated by missense mutations in about 25% of all the human cancers[33]. In the ovary, *KRAS* mutations are more common in mucinous than in nonmucinous ovarian carcinomas[34]. In ovarian serous cancers, *KRAS* is mutated in predominantly in low-grade but not in high-grade serous cancers[35]. Singer's groups performed digital PCR analysis of *KRAS* mutations in low-grade and high-grade ovarian serous carcinomas and found that *KRAS* activating mutations at codons 12 and 13 were prevalent in low-grade and borderline serous cancers, but were completely absent in their high-grade counterparts they examined[32]. In low-grade ovarian serous carcinomas, estimated 27% to 54% of cases harbor mutations in *KRAS* oncogene, whereas in high-grade serous carcinomas, mutation rate of *KRAS* ranges from 0 to 12%[36],[37].

***BRAF:*** The *RAF* family of genes encode cytoplasmic serine-threonine kinases that are activated by *Ras* oncogenes, mediating cellular response to growth stimulatory signals. Somatic missense mutations of *BRAF* have been identified in a variety of cancers including ovarian serous borderline tumor and low-grade serous carcinoma in the RAS-RAF-MEK (mitogen/extracellular signal-regulated kinase)-ERK (extracellular signal-regulated kinase)-MAPK (mitogen-activated protein kinase) pathway[38],[39]. However, mutations of *BRAF* oncogene are rare in invasive high-grade serous carcinoma and in non-serous ovarian tumors[37]. Somatic mutations of *BRAF* occur exclusively within the kinase domain, and in 80% cases constitutively activated *BRAF* is the result of a single amino-acid substitution (V599E)[38]. Davies *et al*[38] reported that *BRAF* mutations in the ovary were largely associated with low-grade serous carcinomas, and not with high grade serous carcinomas. Together, *KRAS* or *BRAF* mutations are present in 68% of low-grade serous carcinomas, whereas they have not been identified in high-grade serous carcinoma[1],[32],[40]. Notably, KRAS and BRAF mutations in serous borderline tumor or low-grade ovarian serous carcinoma are generally mutually exclusive[41].

### Genetic features of high-grade serous carcinoma

***p53:*** *p53* gene is a transcription factor activated by damages to the genome, involved in DNA damage response and activation of apoptosis. *p53* is perhaps the most widely studied tumor suppressor gene in the history of human cancer research. Mutations or overexpression of *p53* occur in 50% to 80% of human ovarian serous carcinomas[42],[43]. Elevated expression of *p53* gene has been shown an independent prognostic factor[44]. Wen *et al*[16] demonstrated that patients with *p53* mutations and/or overexpression had statistically significant shortened overall survival in 105 ovarian carcinoma cases. Mutations and immunohistochemical overexpression of *p53* occur in as much as 95% high-grade[40],[42],[45]; and only 10% to 28% low-grade serous carcinomas[43]. Some studies showed that p53 mutations in low-grade tumors generally appear to occur late in tumorigenesis where they may be involved in tumor progression rather than initiation[42]. Thus, p53 mutation is a signature of high-grade but not low-grade serous cancers.

***Breast cancer susceptibility gene* (*BRCA*) *1/2: BRCA1/2*** play important roles in maintaining genomic stability and act as tumor suppressors in breast and ovarian cancer development. Mutations of *BRCA1/2* are associated with increased susceptibility for breast and ovarian cancer. These mutations increase the risk for developing ovarian cancer by 26% (*BRCA1*) and 10% (*BRCA2*) during a woman's life time[46]. *BRCA1* mutations have a higher incidence in ovarian cancer than *BRCA2* mutations do[47]. Hilton *et al*[48] and Geisler *et al*[49] identified *BRCA1/2* loss-of-function mutations in a majority (84%) of ovarian carcinomas, including somatic cells and germ-line cells. They also demonstrated that loss of *BRCA1/2* function was more frequently present in the sporadic and hereditary high-grade serous carcinomas as compared to the low-grade counterparts. Interactions between *BRCA* genes and *p53* have also been documented. For example, overexpressed *BRCA1* can stabilize *p53*[50].

### Ovarian serous cancer develops through two distinct pathways

In summary, molecular and genetic studies have shown that high-grade ovarian serous carcinomas carry a high prevalence of *p53* and *BRCA* gene mutations but not mutations in *KRAS* or *BRAF* oncogenes; on the contrary, low-grade ovarian serous cancer has a frequency of *KRAS* or *BRAF* mutations but very rarely mutations in *p53* or *BRCA1/2* (***Table 2***), suggesting that high- and low-grade serous carcinomas develop along two distinct pathways. Indeed, morphologically low-grade serous carcinomas show fewer molecular abnormalities by both cytogenetic and single nucleotide polymorphism analysis as compared to those in the high-grade cancers[51]. Additionally, comparative genomic hybridization studies have also demonstrated that whereas low-grade serous carcinomas retained relatively intact genome structures, their high-grade counterparts exhibited extensive genomic instability[52]. Specifically, high grade serous carcinomas showed under-representation of chromosomes 11p and 13q and over-representation of chromosomes 8q and 7p, and low-grade carcinomas showed under-representation of chromosome 12p and over-representation of chromosome 18p more frequently[53]. These marked differences between high-grade and low-grade serous cancers suggest that these two types of ovarian serous cancer, in most cases, arise *via* different genetic pathways[1],[40]. This notion has become increasingly well-received among ovarian cancer clinicians and researchers. In a study conducted in 2005, Singer *et al*[42] referred low-grade and high-grade serous carcinoma to Type I and Type II tumors, respectively. These authors concluded that Type I ovarian serous tumors are low-grade neoplasms that develop in a stepwise fashion from ‘adenoma-borderline tumor-carcinoma’ progression; on the other hand, Type II tumors develop ‘de novo’ from the surface epithelium without morphologically recognizable precursor lesions and grow rapidly[42]. The clinical manifestation, morphologic features and molecular profiles of low-grade and high-grade serous carcinomas are summarized in ***Table 3***.

**Table 2:** The incidence of mutations in *p53*, *BRCA1/2*, *KRAS* and *BRAF* genes in low- and high-grade ovarian serous cancers

	*p53*	*BRCA1/2*	*KRAS*	*BRAF*
Low-grade	10%-28%	Rare	27%-54%	common
High-grade	95%	Common	0-12%	rare

**Table 3:** Difference between low- and high-grade serous carcinomas

	Low-grade (Type I )	High-grade (Type II)
Nuclear atypia	Mild to moderate	Severe
Mitosis	< 12/10HPFs	> 12/10HPFs
Calcifications	Always	50%
With mucin	In most cases	Rare
5-year survival rate	60%	20%
10-year survival rate	30%	5%
Association with serous borderline lesions	Yes	No
Genetic mutations	*KRAS, BRAF*	*TP53, BRCA1/2*

Mechanisms underlying the evolvement of ovarian serous cancer through two distinct pathways are sufficiently explained by the genetics inherent to Type I and Type II ovarian serous cancers. As such, the slow-growing and genomically stable Type I serous cancers result from mutations in the RAS-RAF kinase pathway but not in the “genome guards” *p53* and *BRCA1/2*. Accordingly, Type II serous cancers exhibit widespread genomic instability and develop aggressively because of highly unstable genomic architecture attribute to inactivation of *p53* or *BRCA1/2* tumor suppressors[1],[36],[42]. Combined genetics and morphology are seamlessly unified in defining Type I (low-grade) and Type II (high-grade) ovarian serous cancers. This notion also offers strong support favoring the two-tier grading system developed at the M. D. Anderson Cancer Center over other grading systems, which divides ovarian serous carcinomas into two groups based on histomorphology. The two-tier grading system of serous carcinoma has been widely used in many institutions in the USA, and is expected to gain rapid popularity among medical communities outside the USA as well.
